# Supplementary material for: Unilateral EMG-Guided Botulinum Toxin for Retrograde Cricopharyngeus Dysfunction: A Prospective Clinical and Neurophysiological Study
Source: Toxins (Basel). 2025 Sep 12;17(9):458. doi: 10.3390/toxins17090458 (PMC12474157; doi:10.3390/toxins17090458)
Supplement: Supplementary file 1 [file toxins-17-00458-s001.zip › toxins-3803747-supplementary.pdf]

Supplmentary Material S1. Structured questionnaire for assessing symptom severity and quality of life in R-CPD patients at baseline, 1 month, and 4 months post-treatment.

Symptom-related items					
1. How would you rate your ability to burp?	I burp spontaneously	I need to make an effort but usually succeed	I must use compensatory strategies (e.g., inducing vomiting, abdominal pressure)	I am unable to burp at all	
	0	1	2	3	
2. How often do you burp compared to others?	No difference	Less frequently	Rarely	Never	
	0	1	2	3	
3. Do you hear gurgling noises (e.g., like air bubbles, frog-like sounds) in your throat, especially after meals?	Never	Rarely	Sometimes	Often	Always
	0	1	2	3	4
4. Do you experience abdominal bloating after meals?	0	1	2	3	4
5. Do you experience discomfort or pain in the chest area after meals?	0	1	2	3	4
6. Do you suffer from flatulence?	0	1	2	3	4
7. How would you rate the frequency of your hiccups?	Never/No difference from others	More frequent than others	At least once a day	Dozens of times per day	
	0	1	2	3	
8. Do you experience pain during hiccups?	Never	Sometimes	Often	Always	
	0	1	2	3	
**Quality-of-life items**					
9. Do you avoid certain foods or drinks to prevent symptoms?	Never	Rarely	Sometimes	Often	Always
	0	1	2	3	4
10. Do you avoid social events due to symptoms, whether currently present or to prevent exacerbation?	0	1	2	3	4
11. On a scale from 0 (no impact) to 10 (extremely severe impact), how much do your symptoms impact your daily life?	No impact 0 1 2 3 4 5 6 7 8 9 10 Extremely severe impact				
Total score __/46					
